# In Vivo Basilar Membrane Time Delays in Humans

**DOI:** 10.3390/brainsci12030400

**Published:** 2022-03-17

**Authors:** Marek Polak, Artur Lorens, Adam Walkowiak, Mariusz Furmanek, Piotr Henryk Skarzynski, Henryk Skarzynski

**Affiliations:** 1 R&D Med-El, Furstenweg 77A, 6020 Innsbruck, Austria; 2 Institute of Physiology and Pathology of Hearing, 02-042 Warsaw, Poland; a.lorens@ifps.org.pl (A.L.); a.walkowiak@ifps.org.pl (A.W.); m.furmanek@ifps.org.pl (M.F.); p.skarzynski@ifps.org.pl (P.H.S.); h.skarzynski@ifps.org.pl (H.S.)

**Keywords:** basilar membrane, traveling wave delays, cochlear microphonics, intracochlear electrography, hearing preservation, auditory prostheses

## Abstract

To date, objective measurements and psychophysical experiments have been used to measure frequency dependent basilar membrane (BM) delays in humans; however, in vivo measurements have not been made. This study aimed to measure BM delays by performing intracochlear electrocochleography in cochlear implant recipients. Sixteen subjects with various degrees of hearing abilities were selected. Postoperative Computer Tomography was performed to determine electrode locations. Electrical potentials in response to acoustic tone pips at 0.25, 0.5, 1, 2, and 4 kHz and clicks were recorded with electrodes at the frequency specific region. The electrode array was inserted up to the characteristic cochlear frequency region of 250 Hz for 6 subjects. Furthermore, the array was inserted in the region of 500 Hz for 15 subjects, and 1, 2, and 4 kHz were reached in all subjects. Intracochlear electrocochleography for each frequency-specific tone pip and clicks showed detectable responses in all subjects. The latencies differed among the cochlear location and the cochlear microphonic (CM) onset latency increased with decreasing frequency and were consistent with click derived band technique. Accordingly, BM delays in humans could be derived. The BM delays increased systematically along the cochlea from basal to apical end and were in accordance with Ruggero and Temchin, 2007.

## 1. Introduction

Transduction of sound in human ears is mediated by basilar membrane (BM) waves exhibiting delays that increase systematically with distance from the cochlear base. Numerous studies have been performed in order to measure BM delays. Specifically, postmortem studies in various mammalian species and humans were performed. Delays of BM waves were first measured in the temporal bones of human cadavers by von Békésy [1]. Later, BM delays were measured by several other scientists [2,3]. Another approach to measure BM delays was indirect estimation of BM delays by deriving frequency-specific responses from auditory brainstem responses (ABR; [4,5]) or extracochlear electrocochleography [6], usually by masking with high-pass-filtered noise [7]. Basilar-membrane travelling-wave characteristics have also been studied in humans using transient evoked otoacoustic emissions (OAE; [8,9]) and distortion product OAE [10,11,12]. For OAE techniques, the time taken for sounds of different frequencies to travel from the stimulating/recording probe to the intracochlear site of OAE generation and back again has been elucidated from either direct measurement or by analyzing the phase gradient [9,13]. Some of those estimates have led to the assumption that BM delays are much longer in humans than in common experimental animals [14,15,16]. Ruggero and Temchin (2007), for the first time, estimated in vivo BM delays in human cochlea by correcting postmortem BM data according to the effects of death on BM vibrations in experimental animals [16]. The authors found that death causes increasingly prolonged BM delays by lowering the characteristic frequencies (e.g., the postmortem BM delay was approximately 2 ms longer at 200 Hz) and decreases the delays at characteristic frequencies greater than 2 kHz (e.g., the postmortem BM delay was approximately 0.6 ms shorter at 10 kHz).

Extracochlear electrocochleography has been performed for over 75 years [17]. Deriving frequency-specific responses may lead to the estimation of BM latencies. The recording electrodes are typically placed in the promontory area or in the vicinity of the tympanic membrane. This causes a bias in the source generators coming from the basal part of the cochlea, corresponding to high frequencies, and diminishes the source generator coming from the apical part of the cochlea, corresponding to low frequencies [18]. Extracochlear electrography was performed in individuals with normal hearing and in individuals with a hearing impairment. Cochlear amplifier stops functioning for people with a severe hearing impairment, and as such, the intracochlear signals may be absent or there may be shorter latency responses [6,19].

Nowadays, hearing preservation after cochlear implantation is common even with the use of long electrode arrays [20,21,22,23,24,25,26,27]. By introducing the new cochlear implant technology, in vivo measurement directly from cochlea at various locations is possible in such subjects [28,29,30,31,32]. The feasibility of such recordings was presented at the XXXII World Congress of Audiology in 2014 [33].

Intracochlear recordings may allow for recordings to be made directly from each specific characteristic frequency region. Campbell et al. (2017) measured cochlear microphonic (CM) delays in vivo in 5 cochlear implantees for 500 Hz and 2 kHz; however, these results did not allow for an estimation of BM delays because they were not measured at the characteristic frequency for each region of the cochlea [31]. Bester et al. (2020) measured outer hair cell function in 47 subjects. Within this group, 21 subjects were identified with atraumatic electrode insertion with good hearing preservation postoperatively [34]. The measurement was performed by measuring CM latency shifts across the electrode array contacts being introduced into the scala tympani.

A measuring of BM delays in living humans is of considerable interest due to its fundamental relationship to cochlear physiology. These measurements can provide insights on human cochlear maturation or the possible changes in cochlear mechanics underlying various cochlear-based pathologies. Furthermore, the knowledge of the BM delays can help with the development of future hearing prosthetic devices, utilizing new technologies such as, for example, a piezoelectric artificial basilar membrane (ABM; [35]). Finally, it can help to produce optimal stimulus eliciting evoked auditory responses in human (chirp stimulus).

Our main motivation was to develop and verify a simple method that can be used in humans to measure travelling wave delays. The second motivation was to compare the outcomes with the previous work and helping to address the controversy in the extant literature about the nature of human cochlear tuning compared to that of other animals. The goal of our current study was to access BM delays in vivo via intracochlear electrocochleography across a broad frequency range in a large group of subjects implanted with the medium-long to long electrode array auditory prosthesis. To the best of our knowledge, no such studies have previously been conducted in humans.

## 2. Materials and Methods

### 2.1. Subjects

Sixteen adult subjects (eight females and eight males) with varying degrees of sensorineural hearing loss prior to receiving their cochlear implant (CI) were included in the study. The inclusion criteria included: 1. Ability to give reliable audiometric and psychophysical responses; 2. Electrode insertion angle (cochlea angle measured from the middle of the round window up to the most apical contact of the electrode array) was 460° or higher (equivalent to 500 Hz characteristic location within the cochlea); 3. Subject had functional hearing postoperatively. All subjects were implanted single-sided. Inclusion criteria were not fulfilled in 1 subject where the insertion was 380°. Despite this, the subject was included in the study. All subjects had functional low frequency hearing. Functional hearing was defined as having the ability to use his/her residual hearing and showing a minimum of 5% increased speech understanding with their hearing aids. Speech understanding test was performed prior to and after CI surgery at the time of the study.

The subjects received either a Pulsar, Concerto or Sonata cochlear implant (Med-El Corporation, Innsbruck, Austria) with the following electrode arrays: Flex 20 (*n* = 1), Flex 24 (*n* = 7), Flex 28 (*n* = 2), Flex Soft (*n* = 5), and Standard (*n* = 1). All of these electrode arrays have 12 electrode channels with different total electrode array lengths (L) and distances between the neighboring electrode contacts (D); Flex 20: L = 20 mm, D = 1.5 mm; Flex 24: L = 24 mm; D = 1.9 mm; Flex 28: L = 28 mm; D = 2.1 mm; FlexSoft and Standard: L = 31 mm; D = 2.4 mm. Currently, the only way to measure in vivo BM delays in a living human is to insert the electrode array into the cochlea. Any other attempts are extremely risky for various health issues or may cause complete hearing loss. To minimize the effect of electrode insertion and have the possibility to preserve hearing, we selected electrodes that allows for deep insertion, i.e., reaching the region of 500 Hz and lower (i.e., 250 Hz). For this reason, patients were implanted with the same Flex electrode arrays. Flex electrode arrays are made using the same silicone material and platinum iridium contacts. The only difference among Flex variants is in the length of the array. In patients with larger cochlea, a longer electrode is used, while in patients with smaller cochlea, a shorter electrode is used.

The electrode array was inserted into the scala tympani via the round window for all subjects. The mean age at implantation was 48 years and 4 months (ranged 20–68 years). The mean time of measurements was 13 months (ranged 2–91 months) after CI implantation. All patients provided informed, written consent for their participation in the study and for its dissemination through publication.

### 2.2. Methods

The BM time delay is affected by the biophysical characteristics of the BM and the hydrodynamic environment around the BM. The role of hydrodynamics in the cochlea has been previously discussed in the literature i.e., [36]. Temchin and Ruggero, (2007) reported a difference in delays between healthy cochlea and cochlea investigated postmortem [16]. Therefore, not only the environment, but also proper functioning of cochlear amplifier has an impact on cochlear delays. The patients included in the study were selected to have minimal surgically introduced cochlea trauma. To evaluate the electrode insertion trauma in the scala tympani, the difference between preoperative and postoperative audiograms was assessed. The preoperative audiogram was obtained 1–4 weeks prior to cochlear implantation. The postoperative audiogram was obtained at the time of the study.

**Figure 1:**
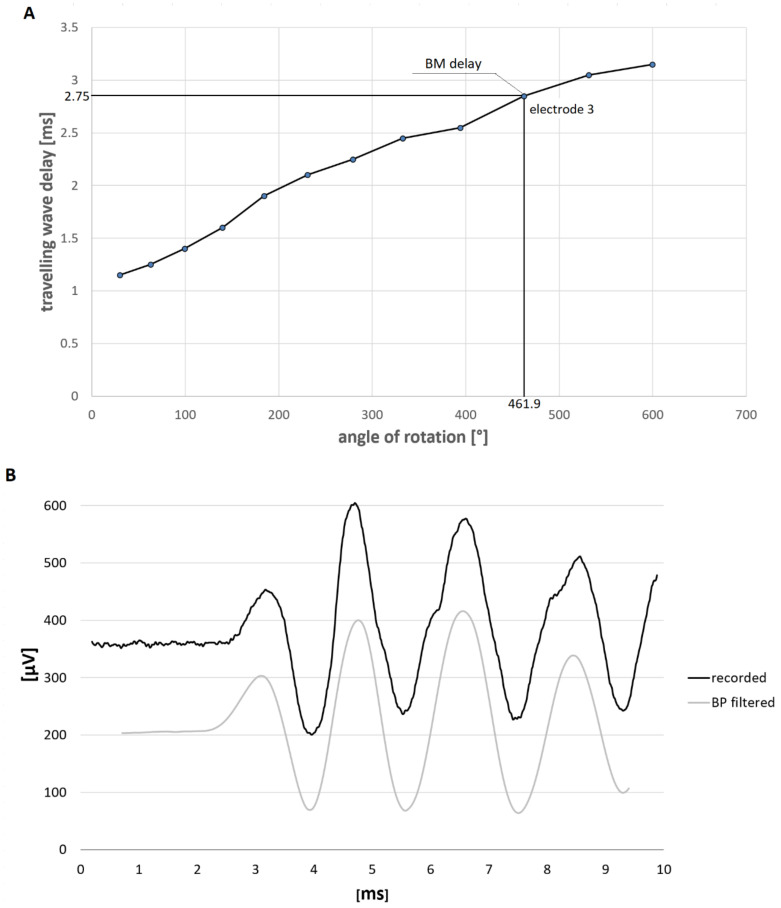

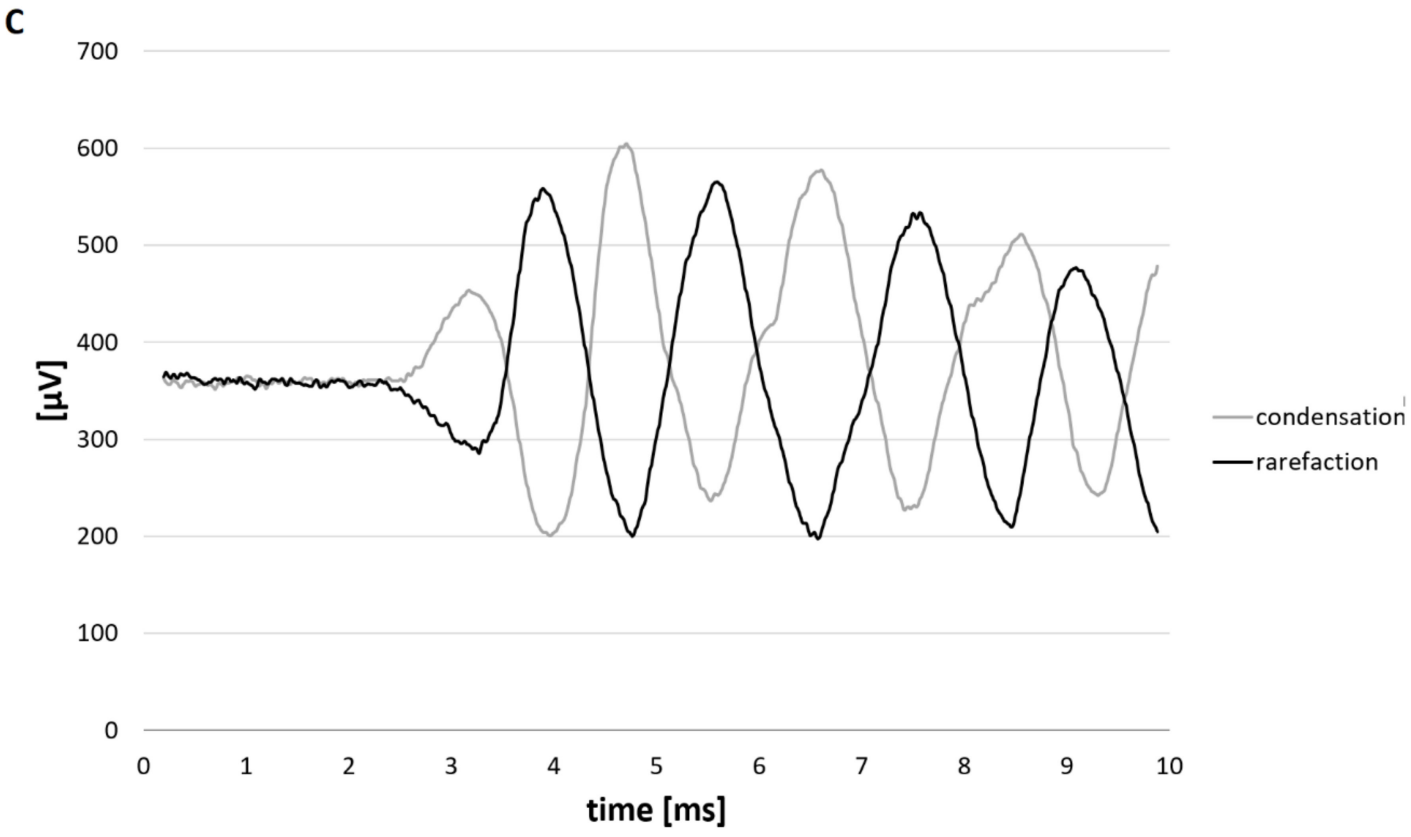
Figure (**A**) Example of an estimated BM delay to a 500 Hz tone burst travelling wave. (**B**) Example of an intracochlear ECochG recordings (black line) and the latency when the CM reached 10% of the maximum amplitude t_10%_ and the latency at the 1st maximum peak t_max_. The grey line is the BP-filtered signal. The stimulus used was a 500 Hz tone pip applied at 0 ms. (**C**) Example of condensation and rarefaction stimulus recordings (grey and black line, respectively).

Postoperative computed tomographic imaging was performed within 1 month after intracochlear electrocochleography (intraocochlear ECochG; [37]) measurements were performed. The electrode location of the intracochlear electrode contacts was derived from the computed tomographic imaging. The Greenwood function was used to derive the characteristic frequency corresponding to the electrode location [38,39]. Estimation of the cochlea angle of rotation and characteristic frequency region was according to [40]. For each participant, the electrodes that were closest to the characteristic frequencies of 0.25, 0.5, 1, 2, and 4 kHz were selected. Each selected electrode was within 35° from the characteristic frequency region. If the electrode insertion did not reach the characteristic frequency of 250 or 500 Hz, e.g., if the insertion angle of the most apical electrode was lower more than 35° at the estimated location of the desired characteristic frequency as calculated using the Greenwood function, then measurements at these frequencies were not performed. This introduced error for implanted electrodes was negligible for a larger sample size (i.e., *n* = 16; mean error was within 5° for each characteristic frequency. Figure 1A explains which electrode is selected for BM delay measurement. The estimated characteristic location for 500 Hz was 457°. The selected recordings for electrode 3 corresponded to an angle of rotation of 461.9°.

### 2.3. Intracochclear Electrococgleography

Recordings of intracochlear EcochG were performed as described by Lorens et al. (2019) [37]. Briefly, intracochlear acoustically evoked potentials were recorded from the electrodes inserted in the scala tympani. Patients were positioned in a comfortable semi-lying position. The acoustic stimulus was presented to the sealed ear canal using the TIP 300 Inserts. The inserts were connected to the Nicolet EDX (Natus Corp., Middleton, WI, USA) which was used for acoustic stimulation. The Research Evoked Potentials (EP) software (Med-El Corp., Innsbruck, Austria) was run from a PC communicating with the MAX Interface (Med-El Corp. Innsbruck, Austria). The MAX interface was connected to the CI via an external coil. When the recording was initiated, the MAX interface triggered the Nicolet EDX which acoustically stimulated the patient. The inserts were calibrated, and the measured levels were calculated in dBHL so that the stimulus levels could easily be compared with the preoperative and postoperative audiograms.

### 2.4. Experiment 1: Tone Burst Stimulation

The following tone pips frequencies (and durations) were used for the acoustic stimulus: 250 Hz (12 ms; 1 + 1 + 1 cycles), 500 Hz (8 ms; 1 + 2 + 1 cycles), 1 kHz (5 ms; 1 + 3 + 1 cycles), 2 kHz (2.5 ms; 1 + 3 + 1 cycles) and 4 kHz (1.25 ms; 1 + 3 + 1 cycles). In order to avoid spectral splatter, the rise and fall times of 1 cycle (1 + *n* + 1 cycles) and Blackman window were used. By applying the Blackman window to the onset and offset of the tone pip, side lobes below 60 dB were reached. This way we prohibited the excitation of a broader section of the cochlea than just the characteristic frequency of the recording location. The duration of the tone pips was chosen to be sufficiently long to identify the CM in the response.

For each subject, the latency of the CM when it reached 10% of the maximum amplitude as this is often measured as the latency of the response (in this study, the onset of CM) was measured. Additionally, the latency of the 1st peak of the CM was measured. Theoretically, the peak latency differs with the onset frequency measured at 10% to a little less than 1/(4 × f) where f is the frequency of the signal. For instance, the difference at 1 kHz is 0.17 ms. Figure 1A shows an example of an estimated BM delay to a 500 Hz tone burst travelling wave.

The CM amplitude was obtained at the onset of the responses, which was testified as being a reliable measure of the hair-cell generator [41]. Both opposing polarities should yield the same latency onset. Therefore, each stimulus was presented with a condensation phase for the first half cycle of the waveform (see the Discussion for the definition of CM and further details). Figure 1B shows an example of responses to the condensation and rarefaction phase stimulus. Prior to these measurements, the noise floor was measured. In order to reach a minimum signal to noise ratio (SNR) of 6 dB, the number of averages varied from 100 to 300 (e.g., larger signals required fewer averages and smaller signals required more averages). The minimum number of averages was set to 100. Depending on the stimuli used, the recording window was increased from 5 to 20 ms. For instance, for the tone pips recordings at 250 Hz pips, the time window was set to 20 ms, while for the tone pips of 4 kHz, the time window was set to 5 ms. Further details on the methodology and control measurements performed prior to the study may be viewed in [37].

The CM was evaluated as a signal of the frequency of the stimulus across a minimum of 3 repetitions. The evaluation was performed in the time and frequency domain. For this purpose, Excel 365 software (Microsoft, Redmond, United States) was used. To evaluate the latencies of the CM, the recorded signal was bandpass (BP) filtered with the cut-off frequencies set to multiples of 0.9 and 1.1 of the stimulating frequency. In order to effectively subtract the CM from the remaining intracochlear potentials, a FIR filter of 120th order with zero delay filtering technique was used. This filter has a very steep filtering slope, e.g., around 720 dB/octave. Additionally, 0.75 ms was subtracted from the values in order to compensate for the acoustical delay in the long tube of the insert TIP-300 earphone (length 255 mm). Note that 0.75 ms follows when a sound velocity 340 m/s is assumed. Figure 1C depicts the example of the recordings and the BP-filtered signal.

### 2.5. Stimulation Level Assessment

Before measuring the intracochlear EcochG, the maximum comfortable level (MCL; note that in standard audiology often MCL expands as “most comfortable level”; here, we defined MCL as the maximum comfortable level in order to identify the comfortable range being equal or lower than MCL; in other words, MCL can be “most comfortable level”, but it cannot be higher than MCL.) was measured for each tone pip. For the selected tone pip, starting from 65 dBHL, the amplitude was increased in 1 dB steps until the subject reached their perceived MCL. The amplitude was decreased in 10 dB steps and again increased in 1 dB steps until the perceived MCL was identified. This was repeated at least 3 times. If the perceived MCL was the same at least 2 of the 3 times, then this value was taken as the MCL. The Wuerzburger loudness scale was used [42] to measure the loudness levels of the MCL, which is based on a 50-point scale, ranging from “not heard” to “too loud”.

All recordings were then performed at the MCL. For the tone pip of 1 and 2 kHz, the MCL was not achieved for three and four of the subjects, respectively. In this case, the participants judged the maximum stimulating level as medium-loud. For the 4 kHz tone pip, it was not possible to reach the MCL for five subjects. In this case, the patients judged the maximum stimulating level as medium-loud (*n* = 2) and soft-medium (*n* = 3). Generally, the MCL was not achieved in participants with an auditory threshold equal to or higher than 100 dBHL. If the MCL was not reached, then the maximum stimulating level was used.

### 2.6. Experiment 2: Level Dependent Recordings

In order to evaluate for the dependency of the latency of the CM response to the stimulus loudness, a subset of 8 participants were tested for the stimulus identified at soft-medium, medium-loud, and at the MCL. Again, to identify loudness levels as soft-medium, medium-loud, or MCL, a 50-point Wuerzburger loudness scale was used. According to the loudness scale, the 20th point of the scale was soft-medium, 30th point was medium-loud, and 40th point was the MCL.

### 2.7. Experiment 3: Derived Method

To validate the above-described BM delay measurement, we accessed BM delays, signal-front delay and group delay, other way. In a subgroup of patients (*n* = 7), we stimulated with the condensation and rarefaction polarity click. The recordings were performed from the electrodes closest to characteristic frequency of 250, 500, 1000, 2000 and 4 kHz. If the electrode array was not inserted into the characteristic region of 250 and/or 500 Hz, the response at these frequencies was not obtained. As the impulse cannot be used in humans, we substituted impulse with a 100 μs duration click. The duration of this click was selected for 2 main purposes: this stimuli duration is typically used in audiology, and secondly, we concentrated on a response within the range of approximately 250 to 4 kHz. The stimulating level was at the MCL. In order to obtain receptor response from the responses, the responses to both condensation and rarefaction polarity click were subtracted. To estimate the signal front delay and group delay at the characteristic frequencies 0.25, 0.5, 1, 2, and 4 kHz, we used a similar technique to that developed by Don and Eggermont (1978) [43]. As we were interested only in the receptor response, the stimulating signal was a click without any additional masked signals. To increase the precision of the recorded data, we incorporated one principal difference. While the derived technique uses recordings from one location, in our case, the recordings were obtained at the location closest to the characteristic frequency. The response at the characteristic frequency was HP-filtered with the cut-off frequency of the characteristic frequency. For every HP filter, a Butterworth filter of 9th order was used. This filter system preferably has a very steep filtering slope, e.g., around 54 dB/octave.

### 2.8. Data Analysis

Statistical analysis was performed using standard parametric techniques (analysis of variance (ANOVA) single factor test and two factor with replication, and an appropriate post-hoc test), comparing the mean latencies in terms of their dependency on the stimulus frequency. Within the subgroup of patients stimulated with various stimulation level (medium-soft, medium-loud, and MCL), analysis was performed with the ANOVA two factor without replication test an appropriate post-hoc test. A criterion of α = 0.05 was applied. For the two-group comparison of means, a two-tailed t-test was chosen. The criterion for statistical power was > 0.9.

## 3. Results

MCL levels measured based on the 50-point Wuerzburger loudness scale were published in our previous work (Lorens et al., 2019). The mean stimulating levels for tone pips at frequencies 0.25, 0.5, 1, 2, and 4 kHz were 100.8 ± 9.1, 105.8 ± 7.6, 112.4 ± 8.5, 115.4 ± 5.2, and 115 ± 1.9 dBHL.

**Figure 2:**
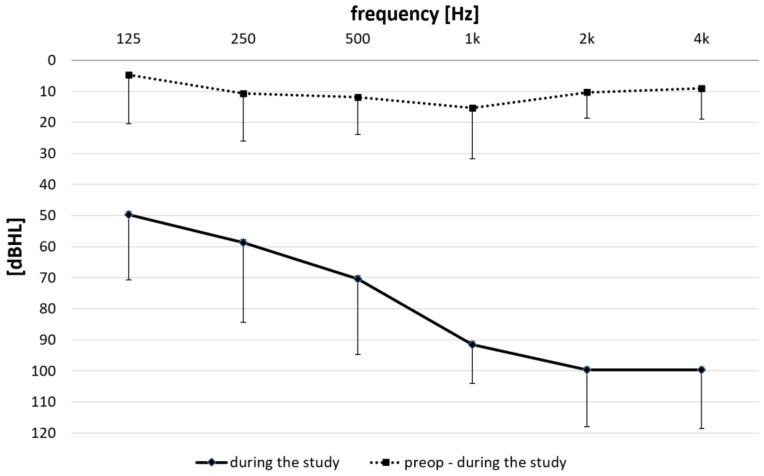
The mean and standard deviation for the postoperative audiogram (squares connected with solid line) obtained at the time of the study and the measured difference between the values obtained immediately before the cochlear implant surgery and at the time of the study (squares connected dotted line) for all subjects (*n* = 16).

Figure 2 depicts the mean postoperative audiogram obtained at the time of the study and the mean difference measured immediately before cochlear implant surgery and at the time of the study for all subjects. The mean pure-tone average (PTA; 250–1000 Hz) shift was 10.6 dB (ranged −1.3–35.0 dB) and the mean PTA at the time of the study was 69.1 dBHL (ranged 27.5–88.5 dBHL). The very low PTA shift suggests that the insertion of the electrode array into the cochlea introduced some hearing-related trauma, but it was relatively small and negligible. Each subject maintained their low-frequency functional hearing, suggesting that a significant portion of inner and outer hair cells retained their functionality.

**Figure 3:**
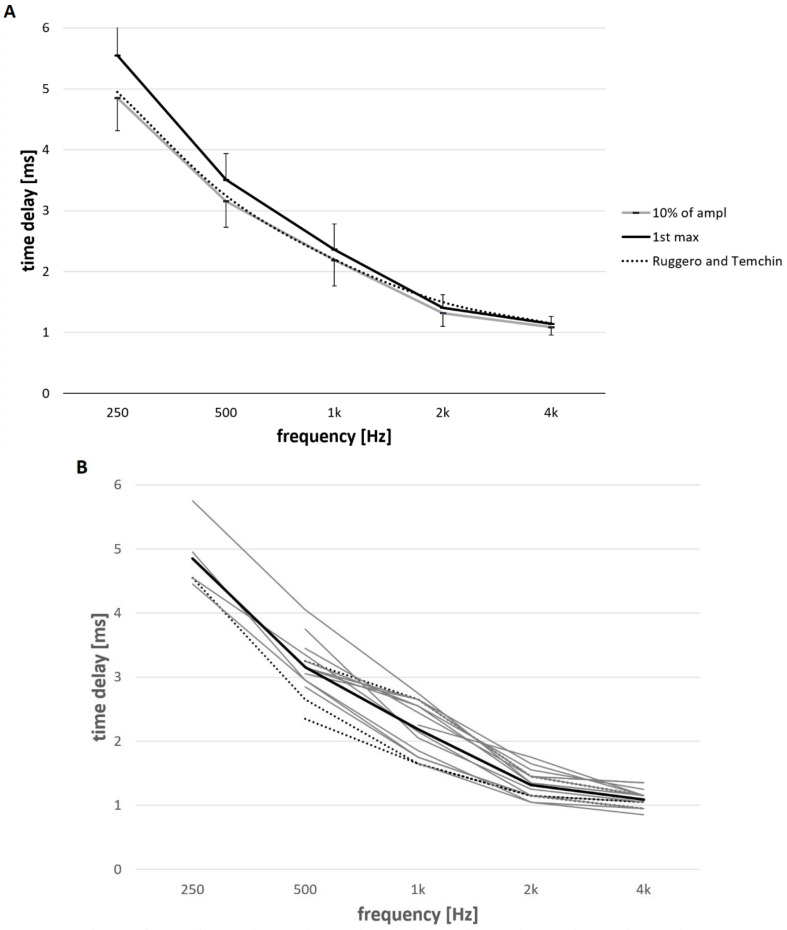

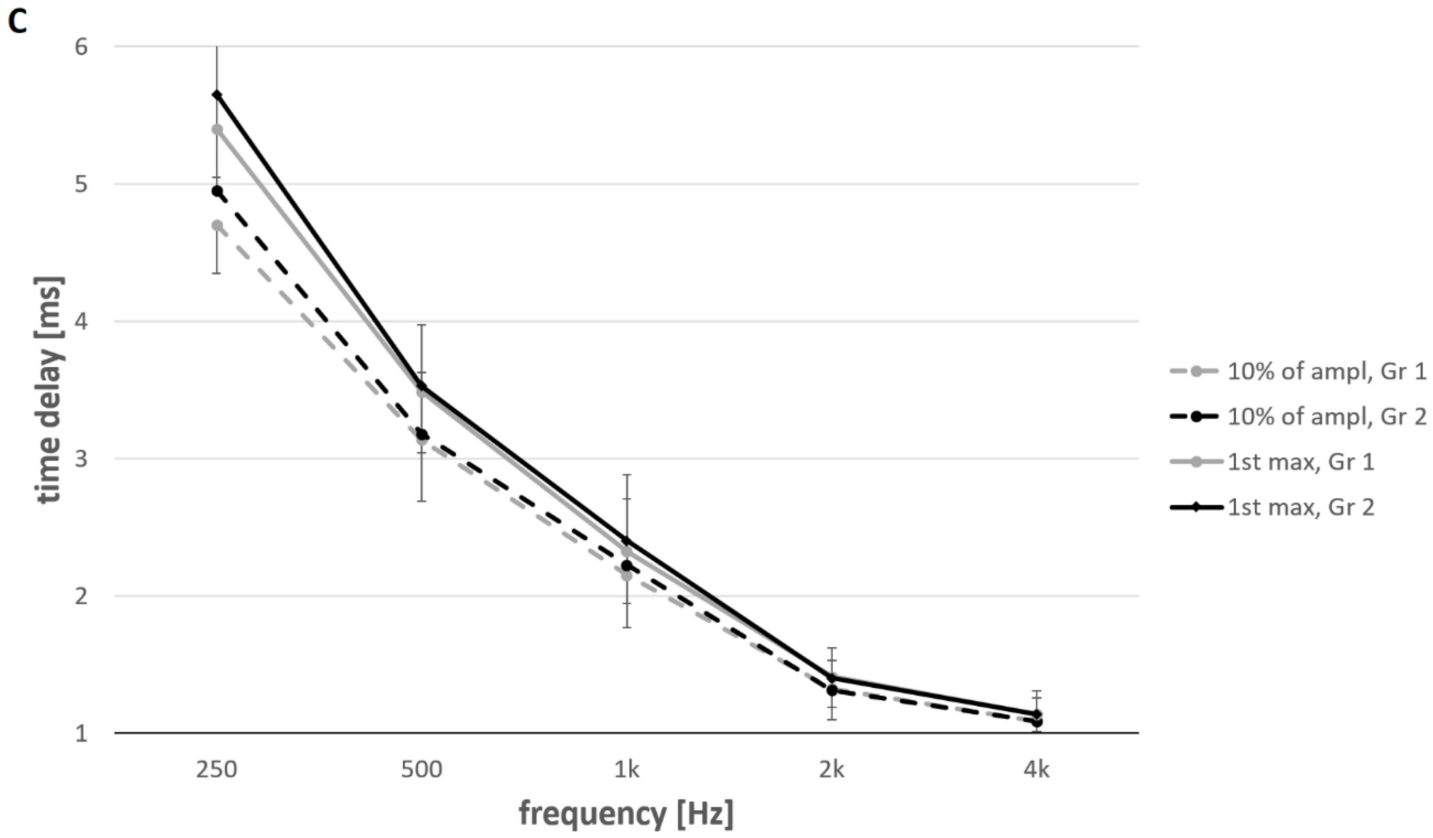
(**A**) The mean and standard deviation of the latency of the 1st CM peak (black line) and the latency when the CM reached 10% of the maximum amplitude (grey line); compared with previous work on basilar-membrane delays by Ruggero and Temchin (2007) [16]. (**B**) Mean (black line) and individual latencies when the CM response reached 10% of the maximum amplitude. The measured latencies of each characteristic frequency are connected with a grey line for each subject (*n* = 6 at 250 Hz; *n* = 15 at 500 Hz; *n* = 16 at 1–4 kHz). Dashed lines show data of three subjects with normal hearing at 125, 250 and 500 Hz. (**C**) Comparisons of the latencies for two different groups. Group 1 contained subjects with hearing better than or equal to the PTA (125–1000 Hz) of 75 dBHL (*n* = 8; *n* = 3 at 250 Hz; *n* = 8 at 500 Hz; *n* = 8 at 1–4 kHz). Group 2 included subjects with hearing worse than the PTA (125–1 kHz) of 75 dBHL (*n* = 8; *n* = 3 at 250 Hz; *n* = 7 at 500 Hz; *n* = 8 at 1–4 kHz).

The electrode array was inserted in the characteristic frequency cochlear region of 250 Hz for six subjects. Furthermore, the electrode array was inserted in the region of 500 Hz for 15 subjects and in the regions of 1, 2, and 4 kHz for all subjects. Intracochlear electrocochleography for each frequency-specific tone pip showed detectable responses in all subjects. Measurements were performed at MCL for each subject. Figure 3A shows the mean latency and standard deviation when the CM response reached 10% of the maximum amplitude and the latency of the 1st peak of the CM. Figure 3B details the individual latencies when the CM response reached 10% of the maximum amplitude; the measured latencies at each characteristic frequency are connected with a grey line that represents each subject. Three subjects with normal hearing at 125, 250 and 500 Hz are marked with dotted line.

An ANOVA single factor test showed that each tested location in the cochlea differed in terms of latencies for various tone pips (α = 0.05; *p* < 0.001; F > 127.2). The post-hoc test showed that the mean difference was significant for each characteristic frequency (two-tailed *t*-test: α = 0.05, *p* < 0.001). Table 1 depicts the mean and standard deviations for the latency when the CM reached 10% of the maximum amplitude and the mean and standard deviations for the latency of the 1st peak of the CM.

Hearing thresholds may correlate to the level of cochlear damage. In order to evaluate if hearing thresholds influence the latencies, the subjects were split into two groups. In order to have approximately equal number of subjects in each group, a criterion of 75 dBHL was used. Group 1 contained subjects with the PTA (125–1000 Hz) less than or equal to 75 dBHL (range 27.5–73.8 dBHL; *n* = 8). The mean PTA of the Group 1 was 55.7 dBHL. Group 2 included subjects with PTA (125–1000 Hz) higher than 75 dBHL (range: 77.5–88.8 dBHL; *n* = 8). The mean PTA of the Group 2 was 80.4 dBHL. Figure 3C shows the comparisons of the latencies for both groups. For the better hearing group (e.g., Group 1), the mean and standard deviation of the latency when the CM reached 10% of the maximum amplitude was 4.70 ± 0.35 at 250 Hz tone pip, 3.14 ± 0.45 at 500 Hz, 2.15 ± 0.38 at 1 kHz, 1.33 ± 0.23 at 2 kHz, and 1.09 ± 0.07 at 4 kHz. Table 2 shows the mean and standard deviation of the latency when the CM reached 10% of the maximum amplitude and the mean and standard deviation of the latency of the 1st CM peak for both the better hearing group (e.g., Group 1) and the poorer group (e.g., Group 2).

Despite the lower mean values for Group 1 at 250, 500, and 1 kHz compared to Group 2, there were no significant differences across all tone pip frequencies between both groups (*p* > 0.05).

**Figure 4:**
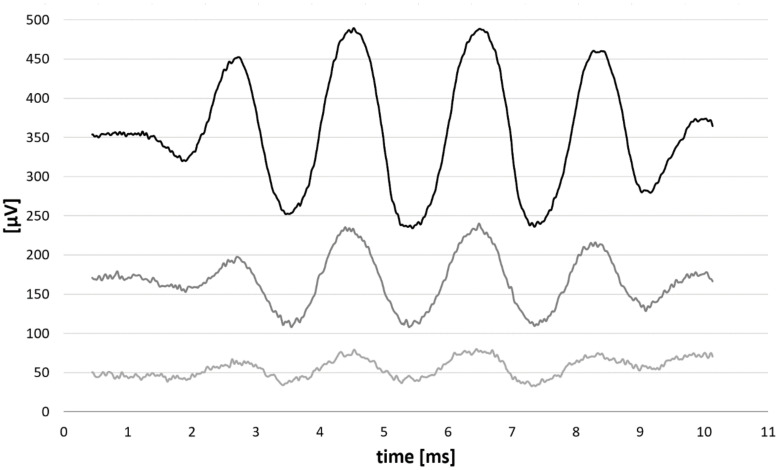
Example of intracochlear ECochG recordings with various loudness stimulus. The loudness perception for the largest response was at the MCL (101 dBHL; black line), the second largest response was at medium-loud (89 dBHL; dark-grey line), and the response with the lowest amplitude was at soft-medium perception (76 dBHL; light-grey line).

**Figure 5:**
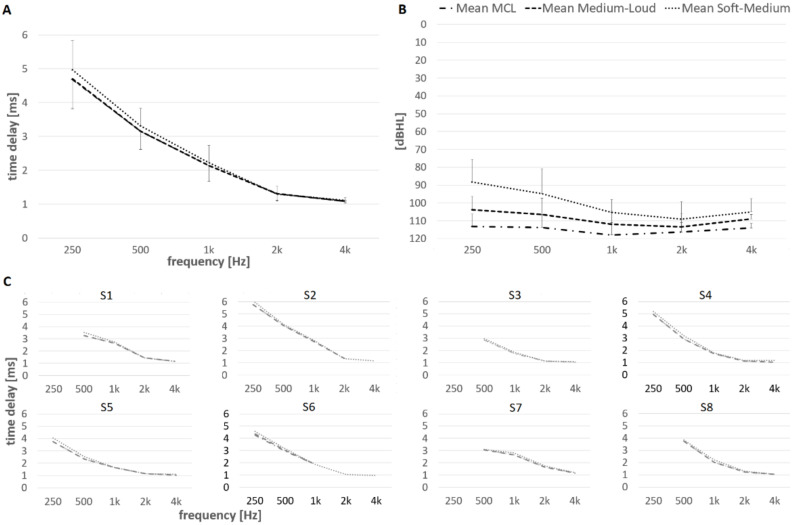
Mean and individual CM onset latencies to stimulus with different loudness perception to tone pip frequencies of 0.25, 0.5, 1, 2, and 4 kHz. (**A**) Mean and standard deviation of latencies when CM reached 10% of the maximum amplitude at soft-medium, medium-loud, and MCL loudness levels; (**B**) Mean and standard deviation of stimulus levels at soft-medium, medium-loud, and MCL loudness levels. (**C**) Individual latencies for each subject (S1–S8) when CM reached 10% of the maximum amplitude at soft-medium, medium-loud, and MCL loudness levels (*n* = 8).

In a subgroup of 8 patients, the onset of CM latencies (e.g., CM reaching 10% of the maximum amplitude) was measured at soft-medium, medium-loud, and MCL loudness levels. Tone pip frequencies of 0.25, 0.5, 1, 2, and 4 kHz were used. Figure 4 depicts an example of a 500 Hz stimulus at three different stimulating levels corresponding to soft-medium, medium-loud, and MCL loudness perception. Figure 5 graphs the mean and individual latencies. As the stimulus level increase, the mean latencies decreased for each stimulating frequency. The mean decrease in the latencies from soft-medium to MCL for the tone pip frequencies 0.25, 0.5, 1, 2, and 4 kHz were 0.29 ± 0.04, 0.16 ± 0.07, 0.09 ± 0.06, 0.05 ± 0.04 and 0.03 ± 0.03 ms, respectively. For the low frequency tone pips (e.g., 250 and 500 Hz), where the mean dynamic range between the soft-medium and MCL loudness perception was largest (mean difference 25.0 and 18.9 dBHL, respectively), the latency differences at soft-medium loudness perception stimulus were largest (mean difference 0.29 and 0.19 ms, respectively). ANOVA two factor without replication test confirmed longer latencies with soft-medium stimuli and shorter with medium-loud and MCL stimuli (α = 0.05; *p* < 0.001; F > 67.0). In both cases the post-hoc test showed that the mean latencies were significant (two-tailed t-test: α = 0.05, *p* < 0.001). No differences in latencies were found between medium-loud and MCL stimuli (two-tailed t-test: α = 0.05, *p* = 0.18). Note that measurements at 250 Hz were only recorded from four patients because only these four participants had an electrode array that was inserted into the 250 Hz region. For the remaining characteristic frequencies, the number of subjects was eight. Furthermore, it was not possible to reach the MCL for the 2 kHz tone pip or the MCL and medium-loud for the 4 kHz tone pip for subject #2. The MCL and medium-loud were not reached for subject #5 for the 2 kHz and 4 kHz tone pips. These missing levels have not been included in the graphs.

**Figure 6:**
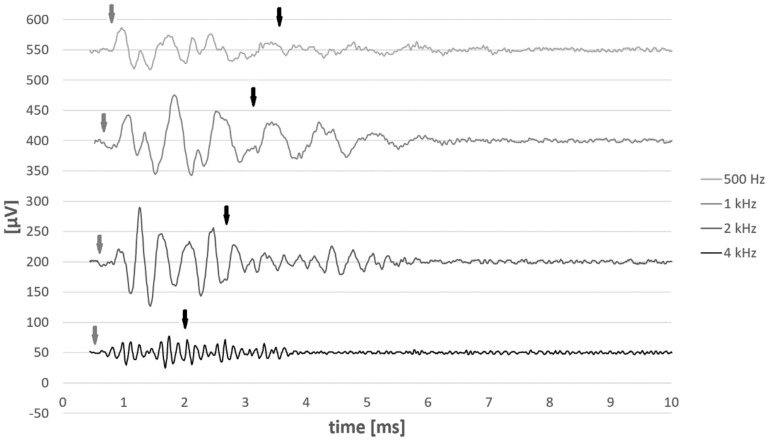
Example of signal-front and BM group delays derived from click response. The recordings were performed from the electrodes closest to characteristic frequency of 0.25, 0.5, 1, 2, and 4 kHz. The responses to condensation and rarefaction polarity click were subtracted, then filtered. The filters used were HP filters with the cut-off frequencies equal to each characteristic frequency (i.e., trace 1 was HP-filtered with 500 Hz cut-off frequency). Grey arrows depict the BM signal front delays, and black arrows depicts the BM group delays.

A subgroup of patients (*n* = 7) was stimulated with the condensation and rarefaction polarity click of 100 μs duration. Figure 6 depicts the example of recordings. In order to obtain receptor response, the responses to both condensation and rarefaction polarity click were subtracted. To estimate the signal front delay and group delay at characteristic frequencies 0.25, 0.5, 1, 2, and 4 kHz, we used a similar technique developed by Don and Eggermont (1978) [43]. In our case, the response at the characteristic frequency was HP-filtered with cut-off frequencies of 0.25, 0.5, 1, 2, and 4 kHz.

**Figure 7:**
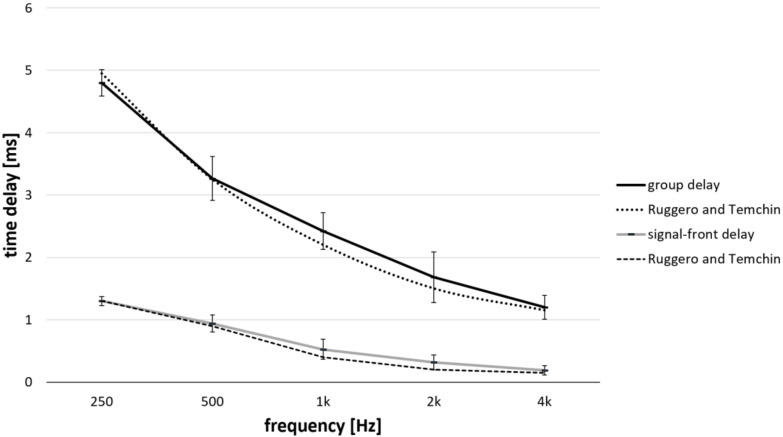
The mean and standard deviation of the signal-front (grey line) and group delays (black line) derived from click response (*n* = 7); compared with previous work on signa-front (dashed line) and group basilar-membrane delays (dotted line) by Ruggero and Temchin (2007).

Figure 7 shows the mean and standard deviations of the signal-front and group delays derived from click response at the MCL. Group delays declined from 4.8 ± 0.21 ms to 1.2 ± 0.19 ms for stimuli ranging from 250 Hz to 4 kHz. Signal-front delays declined from 1.3 ± 0.07 ms to 0.19 ± 0.08 ms for stimuli ranging from 250 Hz to 4 kHz. The measured latencies of each characteristic frequency are connected with a solid line for each subject (*n* = 2 at 250 Hz; *n* = 6 at 500 Hz; *n* = 7 at 1 kHz, *n* = 6 at 2 kHz and *n* = 4 at 4 kHz). Note that measurements at 250 Hz were only recorded from two patients because only these two participants had an electrode array that was inserted into the 250 Hz region. The measurements at 500 Hz were recorded only from two patients because the electrode array did not reach the characteristic frequency of 500 Hz in one participant. In three patients at 4 kHz and one patient at 2 kHz, we were not able to identify any latencies.

## 4. Discussion

### 4.1. Extraction of CM and BM Delay Measurements

This paper elaborates on finding the method that allows us to measure in vivo BM delays in human. For this purpose, we needed to find a reliable method to effectively extract cochlear microphonics from the response that was not contaminated by any other signals, i.e., neurophonics. Several previous studies showed that the evaluation of CM response to tone pips is sufficient with only single stimulus polarity [44,45]. CM has always been considered to have extremely limited clinical use, although much attention has been focused on developing analytical techniques to cancel it from electrocochleographic responses with the aim of extracting the compound action potential (CAP) [45]. More recent studies [46,47] suggest evaluating CM using a technique developed to extract CAP from the ECochG recordings. This includes measurement of the rarefaction and condensation polarity of a broadband stimulus and summating both recordings resulting in CAP recordings [48]. Subtracting both recordings may result in the CM. However, because of the fact that the onset of CM precedes the CAP and neurophonics and we intended to only stimulate single frequencies, it was sufficient to use a single stimulus phase. We did not observe CM saturation in responses during our previous measurements in humans. However, CM amplitudes at frequency tone pips ranging from 250 to 4 kHz at the stimulating levels up to the MCL have been measured at up to 310 uV [37]. On the contrary, extracochlear recordings obtained from animals under anesthesia (e.g., [46]) showed CM saturation, the movement of stereocilia saturated either in one direction (resulting in odd harmonics) or both directions (resulting in even harmonics). The measurements in response to low-frequency tone pips showed contamination by neurophonic potentials generated by the traveling wave at the base of the cochlea [41,46]. These studies showed that the 1st harmonics are solely related to CM for low and mid-low stimuli. This phenomenon of contamination of neurophonics and CM may also occur in humans at very high stimulating levels. From our previous study, if the stimulation level is equal to or lower than the MCL, it can be assumed that the CM is a sinusoid at the stimulus frequency. Nevertheless, the method of subtracting CM from the EcochG signal needs to account for the possibility of contamination of neural responses from the basal part of the cochlea. However, the stimulus only excites the auditory nerve fibers after the respective sensory release of neurotransmitters into the synaptic cleft. The release of transducers takes approximately 1 ms and this delay is independent of the frequency [49]. Therefore, neural response cannot be present at the measurement of onset of CM latency and the first peak of the response for the tested frequency range of 250 to 4 kHz. In case of relatively loud stimuli, i.e., MCL, the tuning curve becomes broader and the neural response from the basal part of the cochlea may precede the hair cell response or neural responses may be contaminated within the first 1 ms of the response. This, however, cannot occur when the input signal is a tone burst and the neural response from the basal part of the cochlea is effectively filtered. To avoid this situation, a bandpass (BP) filter with the cut-off frequencies set to multiples of 0.9 and 1.1 of the stimulating frequency was used. To sum up, the latency of CM can be effectively measured from single stimuli polarity response.

The CM is an alternating current that mirrors the waveform of the acoustic stimulus. It is dominated by the receptor potentials of the outer hair cells of the organ of Corti. Since the CM is proportional to the displacement of the BM, we were interested in measuring the latency of the CM when it reached 10% of the maximum amplitude as this is often measured as the latency of the response. Additionally, to reach higher precision, we measured the latency of the 1st peak of the CM.

Outer hair cells (OHC) are located in the organ of Corti which sits on the BM. They are connected with BM via Deiters cells. OHC stereocilia are bent by a shearing force that occurs when up-and-down movements of the basilar membrane cause it to slide relative to the tectorial membrane, a jelly-like sheet that covers the organ of Corti and in which many OHC stereocilia are embedded. OHCs (and inner hair cells) are depolarized or excited in a graded manner when their stereocilia are bent toward the longest stereocilia, and they are hyperpolarized when stereocilia are bent in the opposite direction. Similar to most other receptor cells, depolarization causes hair cells to release a neurotransmitter substance that activates the neurons connected to them [50]. While OHC diameter keeps a constant value (7 µm), their length regularly varies according to frequency. In the human cochlea, a 25 µm basal OHC is found at a place which codes for 20 kHz; conversely a 70 µm OHC is found apically at the site coding for a very low frequency (<100 Hz; [51]). Considering that the signals are transmitted with the speed of approximately 22 µm/µs (i.e., [52]), CM response starts within 3 µs and thus the CM practically mirrors the BM movement. Therefore, if stimulating with a tone burst of frequencies from 250 to 4 kHz, the onset of CM at the characteristic region may be considered as BM delay of the characteristic frequency.

### 4.2. Effects on BM Delays

To the best of our knowledge, this is the first study to investigate BM delays in the human cochlea using intracochlear ECochG measurements for the broad frequency range 250 to 4 kHz. Ruggero and Temchin (2007) [16] first estimated in vivo BM delays in human cochlea, obtained by correcting postmortem BM data according to the effects of death on BM vibrations in experimental animals. Comparing our mean values (Figure 3A, grey line) with the above-mentioned study, the delays estimated from our tested frequencies (0.25, 0.5, 1, 2, and 4 kHz) were shorter, varying from 0.01 to 0.18 ms. The differences were within 0.2 ms. The outcomes of this study therefore support the fact that that the relationship between postmortem and in vivo delays is similar across species, including humans. Based on this assumption, Ruggero and Temchin were able to estimate in vivo BM delays for living humans on the basis of BM delays in cadavers. Furthermore, signal-front and BM group delays in the cochleae of living humans appear to be similar to the corresponding delays in mammals, and even nonmammalian species, including those in which a BM traveling wave does not exist. The time delays measured in this study may be influenced by several factors such as the presence of the electrode array in the cochlea, individual anatomical parameters of the cochlea, individual cochlear health, and estimated characteristic frequency location. In our study, the recording electrode was selected if the electrode was the closest to and within 35° from the expected characteristic frequency region. The measurements of cochlea delay were performed at MCL.

In a subset of patients, the cochlea delays were measured for soft-medium, medium-loud, and MCL. Interestingly, the performed tests of the stimulus of various loudness perceptions on the subgroup of subjects in this study suggest relatively small variations in time delay (within 0.29 ms). ANOVA two factor without replication test confirmed longer latencies with soft-medium stimuli and shorter latency with medium-loud and MCL stimuli. The data showed a trend between the stimulus and the time delay, whereby the amplitude of the stimulus decreased as the time delays increased and vice versa (Figure 5). This suggests that the cochlea of subjects in this study have active hearing. Similar variations were seen in the literature, i.e., [53], Figure 1, in performed in vivo measurements in animal model. The difference in latency of BM velocity responses to rarefaction polarity clicks in a relatively normal chinchilla cochlea in a limited stimuli range (i.e., 60 dB SPL; this may be considered as soft-to-medium stimuli in normal hearing animals) to loud, (i.e., 90 dB SPL; this may be considered as loud stimuli to normal hearing ears) was less than 0.3 ms. This suggest that the CM latency is dependent on the stimuli loudness similar to BM velocity responses. As demonstrated by Ruggero (1994) [53], as tuning of the cochlear filter broadens, the impulse response times decrease, resulting in shorter cochlear response times.

Another important fact is the hearing level of our study subjects. Ruggero and Temchin (2007) [16], aimed to estimate the delays in a healthy cochlea. The changes in BM delays between postmortem and in-vivo measurements appear to be related to the cochlea amplification mechanism (e.g., the amount by which in vivo sensitivity exceeds postmortem sensitivity at critical frequency). Cochlear amplification depends, especially, on the function of the outer and inner hair cells. While all subjects maintained their functional low frequency hearing, thus suggest that a significant portion of inner and outer hair cells remained functional.

Studies by Liberman and Kiang (1978) [54] have shown that spontaneous activity of auditory nerve fibers after noise trauma is generally unaltered unless there is severe or complete hearing loss for that nerve fiber’s frequency, suggesting that the cochlea and synapses continue to function normally in individuals with moderate hearing losses. This conclusion may imply some loss of cochlear amplification, which does not affect spontaneous activity in the auditory nerve fibers because it is mainly related to normal functioning of IHCs, not to the function of cochlear amplifier. Furthermore, this suggests that in normal hearing subjects we may observe sharp tuning and longer delays to low- and medium-level stimuli.

The Intention of this study was not only to measure BM delays in human, but also estimate in vivo BM delays in normal hearing subjects. Such measurements in normal hearing subjects are not possible as such implantations are not possible due to current CI indications. Our subject population consisted of patients with functional hearing typically suffering from moderate sensorineural hearing loss. In addition, three of the study subjects had normal hearing at 125, 250, and 500 Hz.

Eggermont (1979) [6] and later Don et al., (1998) [43] showed that cochlear hearing losses in humans are similarly accompanied by shorter neural CAP and wave V response times. These outcomes would therefore suggest increased in vivo BM delays in normal hearing subjects.

**Figure 8:**
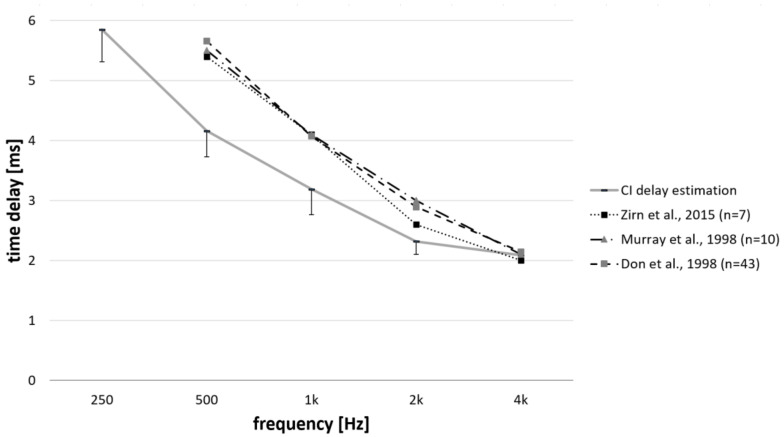
CI delay estimation. The mean and standard deviation for the latency when the CM reached 10% of the maximum amplitude and summation of the time necessary to release neurotransmitters into the synaptic cleft as measured by Temchin et al., 2005 (grey line); compared with previous work on CI delay estimation by Don et al., 1998 [43] (dotted-dashed line); Murray et al., 1998 (dashed line); and Zirn et al., 2015 [5] (dotted line).

Several previous studies showed that in normal hearing subjects wave V latency decreases with the increasing stimulating level for all stimuli frequencies within the speech frequency range, in our case with particular interest between soft-medium (i.e., 60 dB SPL) in normal hearing subjects) to MCL (i.e., 90 dB SPL) in normal hearing subjects). However, the latency decreases when stimulating level increases from 60 dB to 90 dB SPL, is dependent on stimulating frequency and varies from approximately 0.8 ms at 4 kHz to 2.5 ms at 250 Hz [55,56]. Later in the text, it will be discussed that these increased latencies of wave V may not only be influenced by the travelling wave delays but also by the latencies necessary after the process of releasing neurotransmitters into the synaptic cleft up to reaching the total synchronization of neurons (Figure 8).

As we obtained slightly shorter, but similar delays to estimate in vivo BM delays in human, it seems unlikely that the delays will be much larger in normal hearing subjects than in our subjects. It may suggest a clear difference between functional hearing subject’s postmortem cochlea delays. This may be explained by the fact that although CAP and wave V latency shortens in time in subjects with moderate hearing loss; however, these latencies do not seem to shorten at MCL. Therefore, in normal hearing subjects in vivo BM delays may be slightly prolonged. However, according to the measurement in our three normal hearing subjects, it seems that this increase would be lower than the interindividual difference among subjects.

Subjects with moderate hearing loss have their MCL increased by approximately 20 dBHL in comparison to normal hearing subjects. Murray et al. (1998) [56] performed ABR measurements for various tone bursts in normal hearing subjects and patients suffering from moderate sensorineural hearing loss. The study showed that wave V latency decreases with increasing stimulating amplitude independently from the tone burst frequency between 500 to 4 kHz. This was observed for both normal hearing subjects and subjects with moderate hearing loss. Mean wave V latencies for normal hearing subjects were lower than those obtained in patients with moderate hearing loss independent of the tone burst frequency and stimulating amplitude. However, the mean wave V latencies for the group of patients with moderate hearing loss were within the range of wave V latencies obtained in normal hearing subjects. When the 100 dB SPL tone burst stimuli was lowered to 80 dB SPL (i.e., difference in MCL obtained in normal hearing subjects and moderate hearing loss subjects) either in normal hearing subjects or moderate hearing loss subjects, the wave V shifts varied from 0.5 ms for 4 kHz tone burst to 1.0 ms for 500 Hz tone bursts. However, when the tone burst amplitude was 90 dB SPL, the mean wave V shifts between the normal hearing group and moderate hearing loss group, varying from 0.3 ms at 4 kHz tone burst frequency to 0.5 ms are 500 Hz tone burst frequency. The study suggests that the wave V shifts at MCL and therefore, in vivo BM delays for moderate hearing loss subjects and normal hearing subject are comparable.

In our study, Group 1 (*n* = 8) contained subjects with moderate hearing loss (mean low frequency PTA of 56 dBHL). The fact that BM delays of Group 1 are similar, within 0.3 ms of the estimation provided by Ruggero and Temchin (2007) [16], supports that the idea that the relationship between postmortem and in vivo delays is similar across species including humans, although larger cohorts would be needed to verify this. Furthermore, we did not observe any difference in cochlea delay between Group 1 (subjects with better hearing) and Group 2 (subjects with poorer hearing). It is necessary to mention that subjects in this study had various range of hearing abilities (PTA was as low as 27.5 and as high as 88.8 dBHL). Three patients in Group 1 experienced normal hearing at the frequencies up to 1000 Hz, confirming healthy cochlea with the active mechanism functioning without any limitations. However, none of them had the shortest latencies as we would have expected. One explanation may be that the amplitude related differences within our tested intensity range in cochlea delays are smaller than the interindividual differences among our study subjects (Figure 3 and Figure 5). The study outcomes suggest that subjects with functional hearing have BM group delays closer to the healthy cochlea estimates rather than post-mortem cochlea estimates. It would be of further interest to investigate this phenomenon further.

Small delay changes obtained in this study in comparison to the studies measuring CAP or wave V latency changes may be explained by the fact that CM mimics BM displacement with a delay of up to 3 µs, irrespective of frequency. As the CM amplitude is delayed only within 3 µs independent from the frequency from the BM movement, the CM response is practically in phase with BM displacement (consequently, the 1st CM peak will account for π/2 from the BM group delay), unlike for ABR or CAP responses (Figure 8; ABR and CAP delay components are discussed later in the text).

### 4.3. Derived and Other Methods of BM Delays

To validate the above-described in vivo BM delay measurement, we accessed BM delays from click responses by adopting the method proposed by Don and Eggermont, 1978 [43]. As the impulse cannot be used in human, we substituted impulse with a 100 μs duration click. As we are interested only in the receptor response, the stimulating signal was a click without any additional masked signals. By subtracting both the condensation and rarefaction click responses, it is assumed that the neural portion of the response is diminished, but it may not be completely removed [41,46]. However, applying preferably a HP filter with very steep slope and a cut-off frequency of the selected characteristic frequency will guarantee that the phase locked neural component (neurophonics) from the basal part of the cochlea is filtered out and cannot be present during the first 1 ms of the HP-filtered click response. For soft to medium stimuli, neurophonics is of 2nd order of the stimulated frequency, while in louder signals the neurophonics may also be of the 1st order of the stimulating frequency. Either way, the signals can be either of equal or higher frequency than the cut-off frequency of the HP filter. The fact that CM precedes neural response by 1 ms means that we are sure that the HP-filtered and both polarities click-subtracted response does not contain any neural components during the first 1 ms of the response and it does not contain any neural components of neural or receptor generators that are located in the basal part of the cochlea. To increase the precision of the recordings we made one main difference. While the derived technique uses recordings from one location, in our case the recordings were obtained at the location closest to the characteristic frequency. The response at the characteristic frequency was HP-filtered with the cut-off frequency of the characteristic frequency.

There is another possibility to obtain group delays. From the theory of signals and systems, the group delay at any particular frequency is the negative derivate (or slope) of the phase-vs.-frequency function at that frequency (i.e., [57]). This method was not used as it would not reach sufficient precision. Our goal was to develop highly precise method to measure BM delays. For this reason, we attempted to obtain responses at the respective characteristic frequencies. In addition, the recording implant uses sigma-delta modulation of 1.2 MHz sampling rate. Therefore, time domain processing is highly precise.

The mean BM group delay differences among both methods at measured characteristic frequencies of 0.25, 0.5, 1, 2, and 4 kHz were within 0.36 ms, suggesting that both methods lead to an estimation of group delays. Additionally, the tone burst technique (latencies when CM reached 10% of the maximum amplitude) may result in more precise data, as the responses were measurable even in patients with relatively high audible thresholds (i.e., equal to or above 110 dBHL). In several cases when the hearing threshold was not sufficiently low (i.e., below 90 dBHL), we were not able to measure the group delay. This occurred in 3 patients at 4 kHz and in one patient at 2 kHz. Additionally, the signal-front and group delays derived from click response were similar to those estimated by Ruggero and Temchin (2007) [16]. While the maximum mean difference between measured signal-front delays at all measured characteristic frequencies and those estimated by Ruggero and Temchin were within 0.12 ms, the mean differences of BM group delays at all measured characteristic frequencies were within 0.2 ms (Figure 7). Secondly, the literature suggests that the derived method does not necessary lead to correct estimations. The study data support the idea that derived techniques are valid methods even if larger cohorts would be needed to further verify this theory. However, in our case, in order to increase the precision, the recording signal was always obtained at each of the frequency specific cochlear region (0.25, 0.5, 1, 2, and 4 kHz).

### 4.4. Application in Audio Coding Strategies

Obtaining knowledge about the frequency specific time delays within the human cochlea has the potential to improve audio coding strategies for cochlear implants. It was previously shown that people with various degrees of hearing impairment have different time delays caused by the “artificial” processing programs within their hearing aids or cochlear implant audio processors (e.g., [5]). By applying frequency specific time delays to cochlear implant audio processors, it may be possible to achieve time delays that are equal or close to equal for cochlear implant users in comparison to individuals with normal bilateral hearing. According to our experiences, interaural stimulation timing mismatches may result in a limitation in the accuracy of temporal binaural processing. This may be of increased importance as the indication for cochlear implantation continues to expand. Equal time delays may be particularly important for individuals with single-sided deafness that have a cochlear implant on the non-hearing side. Another special interest group may be individuals with normal or near-to-normal low frequency hearing preservation after cochlear implantation [32]. Typically, these groups of individuals have a much higher expectation of their hearing performance in comparison to other cochlear implant candidates. These individuals usually reach the ceiling effect for speech tests in quiet and expect greater improvements with speech in noise test and with spatial hearing abilities.

Note that it is not sufficient to simply implement BM travelling wave delays into cochlear implant audio processors, an additional delay of 1 ms is also needed. While BM delay represents a delay in the travelling wave, BM vibrations in the respective sensory receptor cells are also stimulated and they release neurotransmitters into the synaptic cleft. The stimulus only excites the auditory nerve fibers after this process. The release of transducers is frequency independent and takes approximately 1 ms [49]. This frequency independency has previously also been confirmed in human subjects. The first positive peak P1 of the electrically evoked compound action potential occurs 0.4–0.5 ms after the stimulus is elicited and this is achieved independent of the intracochlear place that is being stimulated (e.g., [58,59]). For electrical stimulation, release of neurotransmitters does not occur and thus this delay should be accounted for in the total time delays.

### 4.5. Previous Works

In this study, the recorded latencies were overall shorter than those estimated by ABR (i.e., [5,43,56]. Zirn and his colleagues estimated CI delays by subtracting the overall wave V latencies from the latency difference between wave V and wave I in 7 single-sided deafness individuals with a cochlear implant on one non-hearing side. Figure 8 shows the comparison of the estimated CI delays as obtained in this study with the previously reported work. The difference may be explained by the fact that the estimation of CI delays by measuring ABR includes the latencies necessary after the process of releasing neurotransmitters into the synaptic cleft up to reaching the total synchronization of neurons, which corresponds to the wave I peak of the ABR. This time delay, however, is frequency dependent and increases with the period of the signal stimulus (Figure 8).

To provide a comparison of the delays, as they were assessed previously, the first peak of the response was measured. Additionally, the latencies at 10% level from the CM amplitude were determined (e.g., onset of CM). Our study only investigated condensation phase stimulus. The fact that the CM is independent of the initial phase of the stimulus rather than reversal of the polarity [60], similar outcomes could be achieved if rarefaction phase stimulus were presented.

Campbell et al. (2017) [31] measured the latencies of the CM in five ears at two frequencies (500 Hz and 2 kHz). The onset of CM was defined as the time at which one of the responses was the first to exceed one quarter of the standard deviation (¼SD) of its signal amplitude evaluated over the duration. Unfortunately, the latencies obtained in that study could not be BM delays as they were not measured at the characteristic frequency for each region of the cochlea. Furthermore, none of the subjects reached insertion depths that represent 500 Hz. The study showed that the hair cells responded to the travelling wave. Specifically, the authors demonstrated that the CM amplitude would increase to the place of maximal displacement of the BM, and the latency would increase with the distance of the recording electrode from the cochlear base, which is in line with our findings. Bester et al. (2020) [34] measured outer hair cell function in 47 subjects with the electrode array inserted such that it included the frequency regions 1 kHz and upwards. None of the subjects had an electrode array inserted in the 500 Hz frequency region. Among those 21 subjects were implanted with atraumatic insertion. The measurement was performed by measuring CM latency shifts across the electrode array contacts being introduced into the scala tympani. Unfortunately, BM delay measurements were not attempted. The authors demonstrated that cochlea regions with functioning hair cells show a detectable latency shift while patients with no measurable hearing demonstrate no latency shift. Furthermore, a significant negative correlation existed between CM latency shifts and hearing thresholds at 1, 2, and 4 kHz when tested with electrodes located at the relevant cochlear tonotopic place.

Our data suggest relatively large variations in the time delays between patients (Figure 3). Nevertheless, the observed interindividual differences in this study are comparable with the interindividual differences reported in other studies that performed ABR in relatively large numbers of humans (e.g., [4,5,56,61]. Therefore, the mean of the data may be adequate.

Shera et al., 2002 [15] used the group delays of stimulus frequency OAEs (SFOAEs) to estimate the critical frequency group delays of BM responses and concluded that BM delays are much longer in humans than in common experimental animals. They based that conclusion on the fact that SFOAE group delays are much longer in humans than in experimental species and on the assumption that the group delay of SFOAEs is equal to twice the group delay of the BM mechanical transfer function evaluated at the cochlear location with critical frequency equal to the stimulus frequency. Harte et al. [8] demonstrated that the ratio of OAE delay, to the BM delay estimated from ABRs, to be 1.9. This is in contradiction to Siegel et al. (2005) [62], who compared SFOAE and BM group delays in experimental animals and found that SFOAEs actually have group delays either similar to or lower than the near-critical frequency BM group delays. This finding may suggest that the assumption may not be valid in other species including human. While in vivo BM measurements in this study and BM delay estimations by Ruggero and Temchin, 2007 [16] were performed at moderate levels, SFOAE delays are estimated at low intensities (30–65 dB SPL). Later studies compared not only SFOAE estimates of delays but also direct physiological measurements from single auditory-nerve fibers in old-world animal model [63,64]. This outcome suggest that the frequency analysis performed by the human cochlea is of significantly higher resolution than found in common laboratory animals.

Other researchers (i.e., [10,65]) estimated BM delays in humans using the group delays of 2f1–f2 distortion product OAEs (DPOAEs) stimulated either by one tone with fixed frequency or with variable frequency. The estimates were based on the assumption that the DPOAE group delays for f2 or f1 sweeps amount to twice the CF group delay of the BM traveling wave. However, other researchers showed on experimental animals this to be not the case [53,66].

Study outcomes in animal models of measurements of BM vibrations (i.e., [67,68,69]), fluid pressure in scala tympani near the BM [70] and responses of auditory nerve fibers [71] have been complemented and interpreted by substantial theoretical and/or modeling work on hydrodynamics [72,73,74,75].

### 4.6. Temporal Synchrony Stimuli Application

Another application of the outcomes of this study may be in adapting the frequency specific delays to create a chirp stimulus or chirps of specific frequency bands. The chirp stimulus is designed to compensate for the time delay in the cochlea and attempts to increase the temporal synchrony between the neural elements that normally are asynchronously activated by a brief stimulus such as a click. Extensive work has been performed to further investigate this. The concept of the chirp was first applied to auditory electrophysiology by Shore and Nutall (1985) [76]. To date, none of the models to create a chirp stimulus were based on in vivo BM delay measurements in humans. The delay models have been applied based on a linear description of the mechanical properties of the cochlea [77], tone-burst ABR latencies [14], stimulus-frequency otoacoustic emission latencies [78], and derived-band ABR latencies [43] and acoustically evoked compound action potential [79]. The gathered in vivo BM delays in this study may be used to help to improve chirp-like stimuli typically used for ABR measurements to further improve the temporal synchrony of neural elements.

## 5. Conclusions

This study investigated BM delays in vivo in humans. CM from characteristic frequency cochlear regions to broad frequency ranges were recorded in 16 study participants with functional hearing. The latencies differed among the cochlear location and the cochlear microphonic onset latency increased with decreasing frequency and were consistent with click derived band technique. The mean values of the frequency specific BM delays were in accordance with Ruggero and Temchin (2007) [16], and do not confirm the long delay times that other measurements have suggested. The outcomes of this study support the fact that signal-front and BM group delays in the cochleae of living humans appear to be similar to the corresponding delays in mammals, and even nonmammalian species, including those in which a BM traveling wave does not exist. This systematic ordering of BM time delays may provide useful information in novel sound coding strategies for an auditory prosthesis or in the development of chirp-like stimulus.

## Figures and Tables

**Table 1:** The mean and standard deviation of the latency of the 1st CM peak and the latency when the CM reached 10% of the maximum amplitude ((*n* = 6 at 250 Hz; *n* = 15 at 500 Hz; *n* = 16 at 1–4 kHz). For the minimum effect of the interest equal to SD, power higher than 0.9 was reached for sample size of 9 and higher was fulfilled for frequencies 0.5, 1, 2, and 4 kHz. For 250 Hz, power of 0.75 was reached.

Frequency (Hz)	250	500	1 k	2 k	4 k
Onset of CM Mean ± SD (ms)	4.85 ± 0.54	3.16 ± 0.43	2.19 ± 0.42	1.32 ± 0.22	1.09 ± 0.13
1st CM Peak Mean ± SD (ms)	5.51 ± 0.58	3.51 ± 0.45	2.36 ± 0.44	1.41 ± 0.23	1.14 ± 0.14

**Table 2:** Comparisons of the latencies for two different groups. Group 1 contained subjects with hearing better than or equal to the PTA (125–1000 Hz) of 75 dBHL (*n* = 8; *n* = 3 at 250 Hz; *n* = 8 at 500 Hz; *n* = 8 at 1–4 kHz). Group 2 included subjects with hearing worse than the PTA (125–1000 Hz) of 75 dBHL (*n* = 8; *n* = 3 at 250 Hz; *n* = 7 at 500 Hz; *n* = 8 at 1–4 kHz). For the minimum effect of the interest equal to SD, power of 0.8 and higher was reached for frequencies 0.5, 1, 2, and 4 kHz. For 250 Hz, minimum power of 0.5 was reached.

Frequency (Hz)	250	500	1 k	2 k	4 k
Group 1 Onset of CM Mean ± SD (ms)	4.70 ± 0.35	3.14 ± 0.45	2.15 ± 0.38	1.33 ± 0.23	1.09 ±0.07
Group 1 1st CM Peak Mean ± SD (ms)	5.40 ± 0.38	3.49 ± 0.47	2.33 ± 0.41	1.41 ± 0.25	1.14 ± 0.07
Group 2 Onset of CM Mean ± SD (ms)	4.95 ± 0.69	3.18 ± 0.45	2.23 ± 0.48	1.31 ± 0.22	1.09 ± 0.17
Group 2 1st CM Peak Mean ± SD (ms)	5.65 ± 0.71	3.53 ± 0.47	2.40 ± 0.47	1.40 ± 0.22	1.14 ± 0.17
